# Largely ignored—but pathogenetically significant: ambient temperature in rodent sepsis models

**DOI:** 10.1186/s40635-024-00693-w

**Published:** 2024-11-14

**Authors:** Reinhard Bauer

**Affiliations:** Institute of Molecular Cell Biology, Jena University Hospital, Hans-Knöll-Strasse 2, 07745 Jena, Germany

## Background

It has long been known that one of the important symptoms of life-threatening infections, including sepsis, is a severe disturbance of thermoregulation [1]. In addition, although it has been studied many times, the question of whether treatment of thermoregulatory disorders in sepsis is beneficial for course of the disease or not has still not been resolved [2, 3]. However, a mechanistic approach to describe the pathogenetic role of thermoregulation in course and outcome of sepsis and to clarify it in more detail in preclinical, translational sepsis research has so far been largely neglected [4]. It is, therefore, a great achievement that da Costa et al. (this issue) [5] investigated the effect of influencing thermoregulation by modulating the ambient temperature (Ta), comparing septic rats with thermoneutral Ta (Ta = 28°C) versus septic rats with hypothermic Ta (Ta = 22°C) on course and outcome of an abdominal infection in the CLP model of varying severity. The result regarding the primary outcome was impressive: reducing Ta by 6°C improved the survival rate in severe abdominal sepsis by 80%. In addition, the study demonstrated a clear dependence of probability of survival on severity of sepsis. However, the pathomechanisms underlying this impressive finding remained mainly elusive. The only reliable finding was that all animals that did not develop a fever or whose fever remained ≤ 40°C survived the early phase of abdominal sepsis (observation period 12 h). All animals with fever > 40°C died. In contrast, the blood prostaglandin E2 levels, which are responsible for at least the early phase of inflammation-induced fever, were largely consistent in both test groups. The same applied to the measure of pro- and anti-inflammatory cytokines.

Less surprising were the minimal differences in protein content of uncoupling protein 1 in the interscapular brown adipose tissue. The latter obviously reflects one of the weaknesses of the study: the rats examined were kept under "controlled ambient temperature of 23 ± 1°C" until 2 days before the start of the experiment, i.e., rats were cold-adapted. Furthermore, 2 days under thermoneutral Ta were obviously too short to allow adequate physiological acclimatization. This reveals a fundamental problem of preclinical translational-oriented sepsis research: in the vast majority of experimental studies—especially on rodents—the inherent peculiarities of thermoregulation as one of the fundamental phylogenetically decisive physiological adaptations in mammals are largely ignored. The sepsis experiments are carried out on cold-adapted animals and pathogenetic links are then made to humans suffering from sepsis, who in the vast majority of cases have lived under thermoneutral conditions. A rethinking is required and the recommendations on good scientific practice in preclinical (translational) sepsis research should also incorporate it in the near future. It goes without saying that the pathogenetic significance of cold-adapted and subsequently cold-stress abdominal sepsis and its stratification can only be determined empirically. Therefore, studies such as those by da Costa et al. (this issue) are very welcome.

## Data Availability

Data supporting the reported results can be requested from the corresponding author.
